# RDW-YOLO: A Deep Learning Framework for Scalable Agricultural Pest Monitoring and Control

**DOI:** 10.3390/insects16050545

**Published:** 2025-05-21

**Authors:** Jiaxin Song, Ke Cheng, Fei Chen, Xuecheng Hua

**Affiliations:** School of Computer, Jiangsu University of Science and Technology, Zhenjiang 212100, China; 231210702101@just.edu.cn (J.S.); 231110703104@just.edu.cn (F.C.); huaxuecheng8888@gmail.com (X.H.)

**Keywords:** pest detection, deep learning, YOLO11, smart agriculture

## Abstract

Due to target diversity, life-cycle variations, and complex backgrounds, traditional pest detection methods often struggle with accuracy and efficiency. This study introduces RDW-YOLO, an improved pest detection algorithm based on YOLO11, featuring three key innovations. First, the Reparameterized Dilated Fusion Block (RDFBlock) enhances feature extraction via multi-branch dilated convolutions for fine-grained pest characteristics. Second, the DualPathDown (DPDown) module integrates hybrid pooling and convolution for better multi-scale adaptability. Third, an enhanced Wise-Wasserstein IoU (WWIoU) loss function optimizes the matching mechanism and improves bounding-box regression. Experiments on the enhanced IP102 dataset show that RDW-YOLO achieves an mAP@0.5 of 71.3% and an mAP@0.5:0.95 of 50.0%, surpassing YOLO11 by 3.1% and 2.0%, respectively. The model also adopts a lightweight design and has a computational complexity of 5.6 G, ensuring efficient deployment without sacrificing accuracy. These results highlight RDW-YOLO’s potential for precise and efficient pest detection in sustainable agriculture.

## Simple Summary

Pests pose a significant threat to agriculture, leading to economic losses and jeopardizing global food security. Nevertheless, effective pest detection in complex agricultural environments remains challenging due to morphological variations between larval and adult stages and high inter-species similarity. In this study, we propose RDW-YOLO, an enhanced pest detection algorithm based on the YOLO11 framework. The proposed model enhances detection accuracy and computational efficiency by incorporating advanced feature extraction modules and an optimized loss function. Experiments on the improved IP102 dataset confirm that RDW-YOLO surpasses existing methods, offering a robust and practical solution for real-time pest monitoring in intelligent agriculture.

## 1. Introduction

The rapid advancement of agriculture has brought increased attention to the persistent challenge of crop pests and diseases, which pose substantial threats to global agricultural productivity and food security. Pests exhibit diverse morphological characteristics throughout their life-cycles and can severely damage crops by feeding on plants and transmitting pathogens. This damage can result in significant yield reductions, complete harvest failures, and severe economic losses for farmers [1]. Moreover, certain pest species exhibit high reproductive rates and remarkable environmental adaptability, leading to rapid proliferation and significant ecological risks, undermining environmental sustainability and agricultural stability [2]. Consequently, accurate and efficient pest detection across various growth stages is critical for ensuring food security and enhancing agricultural productivity.

Pest detection methods are generally classified into manual feature-based techniques and deep learning-based approaches. Traditional methods rely on handcrafted features such as color, shape, and motion, often supplemented by image preprocessing techniques and classifiers like Support Vector Machines (SVMs) and k-Nearest Neighbors (KNNs) [3,4,5]. However, these approaches are highly susceptible to variations in environmental conditions, including complex backgrounds, fluctuating lighting conditions, and high intra-class variability among pests. Consequently, they lack the robustness and real-time detection capabilities needed for modern agricultural applications.

**Figure 1:**
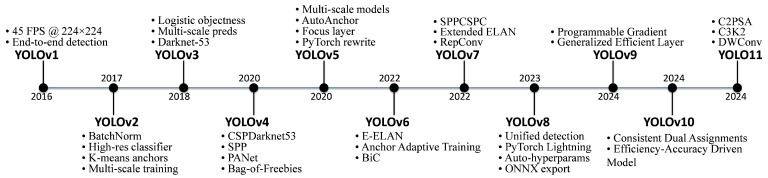
Overview of improvements in YOLO versions from YOLOv1 to YOLO11.

Deep learning techniques have significantly improved pest detection performance by enabling robust feature extraction [6]. Two-stage object detection frameworks achieve high detection accuracy but are computationally intensive. In contrast, single-stage detectors, such as those in the YOLO (You Only Look Once) family, prioritize inference speed, making them ideal for real-time pest monitoring. Successive iterations from YOLOv1 to YOLOv10 have continuously improved feature fusion, network architecture, and loss function optimization, establishing YOLO as a leading approach in pest detection [7,8,9,10,11,12,13,14]. The improvements across different YOLO versions are illustrated in Figure 1. However, challenges such as complex backgrounds, occlusions, and high target similarity still lead to misdetections, particularly in cluttered environments.

With the rapid development of agricultural intelligence, pest detection technologies have progressively evolved toward higher precision, real-time responsiveness, and environmental adaptability. To address the performance degradation under dynamic environmental conditions, Wang et al. [15] proposed an adaptive continual test-time domain adaptation strategy, significantly enhancing model robustness against variations in illumination and weather. Concurrently, Shoaib et al. [16] provided a comprehensive review of recent advances in deep learning applications for plant disease and pest detection, highlighting persistent challenges in data diversity, detection accuracy, and practical deployment efficiency. To expand the application scope and improve large-scale monitoring capabilities, Lee et al. [17] explored the integration of remote sensing technologies with artificial intelligence, enabling wide-area, high-precision pest surveillance. Furthermore, Yu et al. [18] introduced cross-domain data augmentation with domain-adaptive language modeling, offering promising solutions for data scarcity issues in pest detection tasks. For practical deployment, Chen et al. [19] developed an embedded drone system empowered by deep learning, enabling precise identification of fruit tree pests and targeted pesticide spraying, thus advancing the implementation of intelligent agricultural machinery.

Building upon these foundations, various innovations have been introduced to further address the limitations in complex environments, small-object detection, and multi-target processing. For instance, Dong et al. [20] proposed PestLite, integrating spatial pooling and attention mechanisms to optimize multi-scale detection while reducing model complexity. Tian et al. [21] introduced MD-YOLO, integrating DenseNet and adaptive attention to enhance detection accuracy. Yu et al. [22] optimized YOLOv8 for small-target detection using attention modules, while Wei et al. [23] introduced AEC-YOLOv8n to enhance multi-target detection in complex agricultural environments. Wu et al. [24] further improved real-time monitoring by integrating YOLOv5 with DeepSort, achieving 94.3% tracking and 94.1% counting accuracy. Meanwhile, Yang et al. [25] developed Maize-YOLO for maize pest detection, attaining 76.3% mAP on the IP102 dataset. Kang et al. [26] enhanced small pest detection under complex conditions using a Coordinate Attention-Based Feature Pyramid module. Additionally, multimodal data, such as infrared and spectral imaging, have been explored to improve detection robustness in challenging environments [27,28,29]. These advancements collectively refine pest detection models, addressing key challenges in precision, scalability, and real-world deployment.

Despite these advancements, effective pest detection models must be capable of identifying pests in their early larval stages, handling various background environments and different lighting conditions, and ensuring an optimal trade-off between accuracy and computational efficiency, especially in resource-limited environments. Moreover, improving generalization through data augmentation and advanced feature extraction techniques is crucial to mitigating the impact of imbalanced data distributions.

This study introduces RDW-YOLO, an enhanced pest detection algorithm built upon the YOLO11 framework. YOLO11 offers significant improvements in network architecture and feature extraction, specifically designed to address the morphological variations of pests at different life stages and detect fine-grained features. RDW-YOLO further extends YOLO11 by incorporating multi-scale feature fusion modules and optimized bounding-box regression strategies, thereby enhancing the detection of subtle pest characteristics and diverse species.

Specifically, our key contributions are summarized as follows:

1. The RDFBlock module is seamlessly embedded into the network architecture, augmenting the model’s capability to capture and represent intricate pest characteristics. Incorporating multi-branch expansion convolution effectively improves the detection accuracy for diverse targets.
2. The downsampling module DPDown is optimized by combining hybrid pooling and convolution operations. This significantly improves the efficiency of feature extraction and enhances the model’s adaptability to target features at different scales.
3. The loss function is improved, and the WWIoU is innovatively proposed. Fusing the Normalized Wasserstein Distance (NWD) and Wise-MPCIoU mechanisms enhances the matching accuracy of prediction frames and the location regression ability, thereby improving the overall performance of pest detection.

## 2. Materials and Methods

### 2.1. Datasets

The IP102 dataset, initially introduced by Wu et al. [30] in their pioneering study on large-scale agricultural pest detection, serves as a comprehensive benchmark for pest identification tasks. It encompasses 102 distinct pest categories and consists of approximately 22,284 images, systematically capturing the full developmental trajectory of pests, from larvae to adult stages. The dataset is publicly available on GitHub (https://github.com/xpwu95/IP102, accessed on 10 September 2024).

**Figure 2:**
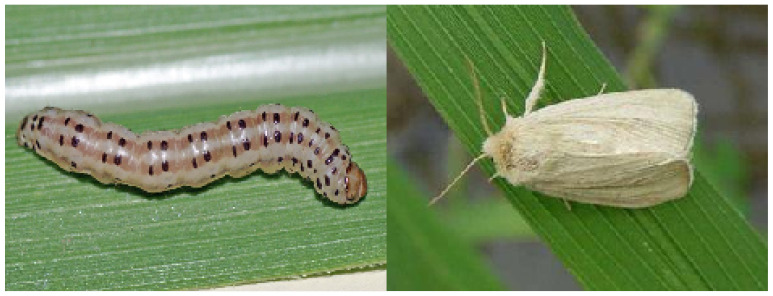
Intra-category differences caused by pest life-cycle changes.

**Figure 3:**
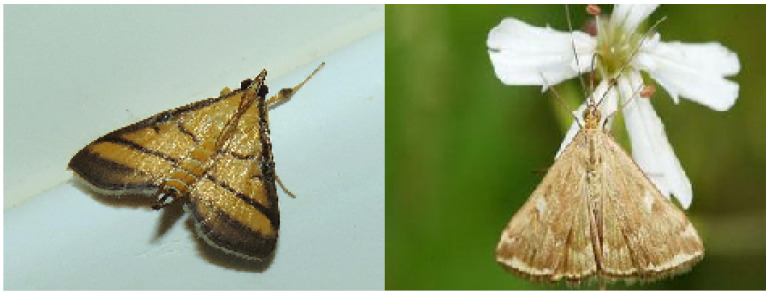
High feature similarity between categories.

This dataset possesses several distinguishing characteristics. It includes a refined hierarchical classification system and a natural long-tailed distribution. Additionally, it exhibits high feature similarity between categories, as shown in Figure 2, and significant intra-category differences caused by pest life-cycle changes, as shown in Figure 3. These features highlight the diversity of pest species while enhancing the complexity and applicability of the dataset, making it an invaluable resource for pest detection research.

**Figure 4:**
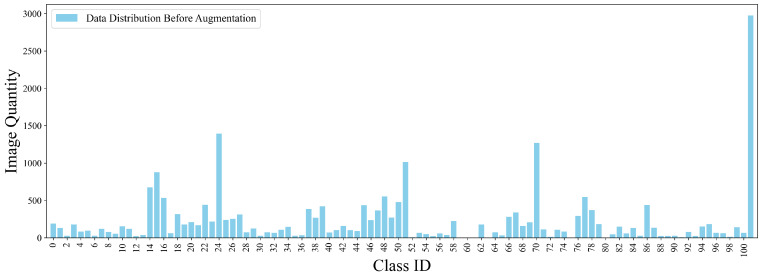
Initial category distribution in the original IP102 dataset.

One notable aspect of the dataset is the pronounced imbalance in the varying distribution of images among categories. As shown in Figure 4, certain categories, such as category 24 (*ants*), category 70 (*blind stink bugs*), and category 101 (*leafhoppers*), contain a significantly larger number of images compared to others. Conversely, categories such as category 2 (*paddy stem maggot*), category 4 (*yellow rice borer*), and category 75 (*Phyllocoptes oleiverus ashmadea*) suffer from a severe lack of samples.

To mitigate data imbalance in the dataset, an additional 300 images were collected and manually annotated from online sources, specifically targeting categories with limited representation. These images were annotated using the Labelme tool, ensuring visual consistency with the predefined categories in the IP102 dataset. Furthermore, various data augmentation techniques were employed to enhance the quantity and diversity of training samples. The augmentation methods included brightness adjustment, contrast enhancement, color saturation amplification, Gaussian blurring, image rotation, and detail enhancement. These techniques expanded the dataset and improved its variability, thereby strengthening the model’s generalization performance. As a result, the total number of images in the dataset increased by 2874.

**Figure 5:**
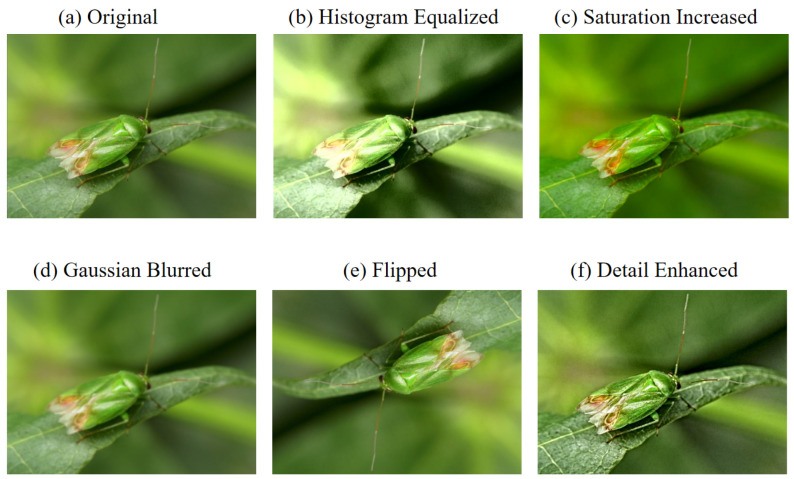
Examples of data augmentation applied to category 57 (*Apolygus lucorum*): (**a**) original; (**b**) histogram equalized; (**c**) saturation increased; (**d**) Gaussian blurred; (**e**) flipped; (**f**) detail enhanced.

For instance, category 57, corresponding to the (*Apolygus lucorum*), originally contained a limited number of samples. Through the implementation of the aforementioned augmentation strategies, the number of images in this category was increased to 116. This augmentation process significantly contributed to mitigating the class imbalance problem in the dataset and is illustrated in Figure 5.

**Figure 6:**
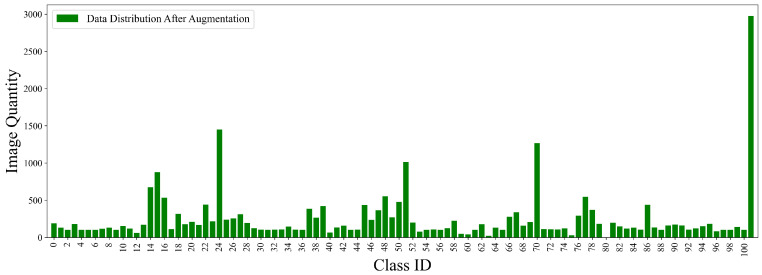
Category distribution in the enhanced IP102 dataset after data processing.

This augmentation expanded the dataset to a total of 25,158 images. Figure 4 illustrates the sample distribution across categories prior to data enhancement, whereas Figure 6 showcases the distribution post-enhancement, alongside the spatial distribution of target locations and object sizes in the augmented dataset. Finally, the dataset was partitioned into training, validation, and test subsets in a ratio of 70:20:10 to ensure a standardized evaluation protocol.

### 2.2. RDW-YOLO

**Figure 7:**
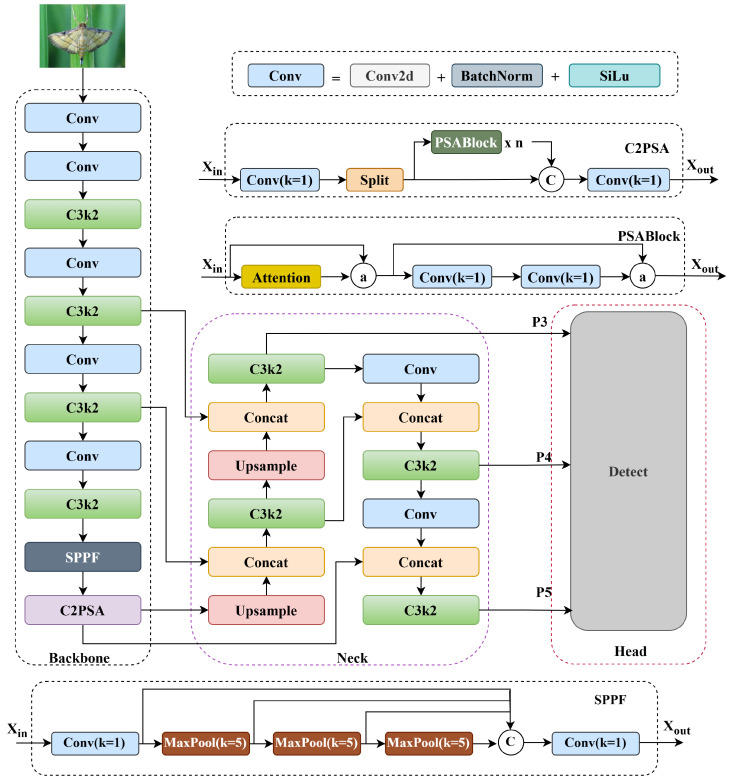
Network structure of YOLO11.

YOLO11 introduces key optimizations over YOLOv8, focusing on network structure and performance improvements [31]. It features an enhanced Backbone, improved Neck, and efficient Head. The Backbone integrates C3k2 and C2PSA modules to strengthen feature extraction and representation. The Neck replaces the C2f block with C3k2 to improve feature aggregation, while C2PSA enhances spatial attention, aiding in the detection of small or occluded objects. In the Head, multiple C3k2 blocks support better multi-scale feature processing, boosting prediction accuracy. YOLO11 also reduces model parameters, ensuring faster inference and compatibility with resource-constrained devices. Its overall architecture is shown in Figure 7.

Despite these advancements, YOLO11 still faces several limitations in specific application scenarios. For instance, its detection performance in complex backgrounds, such as dense foliage or cluttered agricultural scenes, remains suboptimal due to challenges in distinguishing pests from visually similar background textures. Additionally, while YOLO11 balances accuracy and efficiency, its lightweight design limits its capacity to capture highly complex or fine-grained features, limiting its effectiveness for detecting extremely small or camouflaged pests.

**Figure 8:**
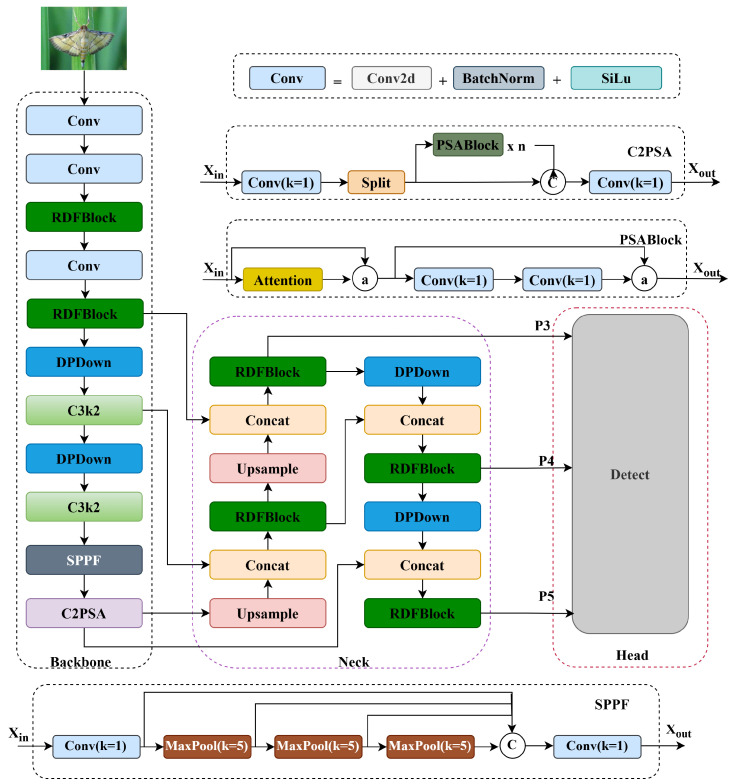
Network architecture of RDW-YOLO for pest detection.

To address these limitations, this paper proposes an enhanced YOLO11 architecture aimed at developing a more robust and efficient pest detection model. The optimized network structure is presented in Figure 8. The improved model introduces three key enhancements: first, integrating the RDFBlock module into the backbone network significantly enhances feature extraction by employing multi-branch expansion convolutions, thereby improving the model’s ability to capture complex target features. Second, incorporating the DPDown module in the downsampling process combines hybrid pooling with convolution operations, increasing feature extraction efficiency and improving adaptability to multi-scale targets. Finally, the original loss function is replaced with WWIoU, which offers more accurate matching between predicted bounding-boxes and ground-truth annotations, leading to improved position regression accuracy. These enhancements substantially elevate the overall detection performance of the model, particularly in complex and challenging environments.

### 2.3. Reparameterized Dilated Fusion Block

In the YOLO11 model, the C3K2 module serves as a critical component of the feature extraction network. It is designed to achieve efficient information interaction and fusion between feature channels through its bottleneck structure and cross-layer connections. The model’s core features include grouped convolutions and residual connections, which improve feature representation and optimize computational resource efficiency. However, the traditional C3K2 module, constrained by a fixed receptive field, exhibits limitations when handling complex background information, multi-scale target detection, and small target recognition.

**Figure 9:**
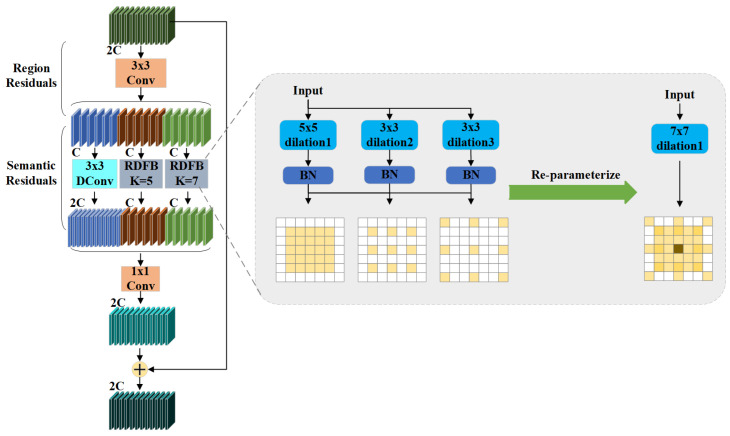
Architecture of the improved RDFBlock module.

To address the aforementioned limitations, this paper introduces an enhanced module termed the Reparameterized Dilated Fusion Block (RDFBlock). This design builds upon the core idea of the Dilated Reparameterization Block, a pivotal architectural innovation initially proposed in UniRepLKNet [32]. The RDFBlock enhances the representational capacity of large-kernel convolutional layers by integrating multiple parallel small-kernel convolutions with varying dilation rates. Such a configuration enables the model to effectively capture sparse spatial patterns and substantially enlarge the receptive field. During the training phase, the outputs from the large-kernel convolution and the parallel dilated convolutions are fused to leverage multi-scale contextual information. Subsequently, these components are structurally reparameterized in the inference phase into a single equivalent large-kernel convolutional layer, thereby minimizing computational complexity without compromising performance. This reparameterization strategy ensures that only one convolution operation is required at inference time while retaining the expressive benefits acquired from diverse receptive fields during training. By incorporating dilated convolution and reparameterization techniques into the original C3K2, the RDFBlock module significantly enhances receptive field size and multi-scale feature modeling capabilities. The architectural design of the RDFBlock module is depicted in Figure 9.

In designing expansion convolutions, the area of influence of the convolutional kernel is defined by the dilation rate *r*, which is given by the following:

(1) R=k+(k−1)×(r−1)

Let *R* represent the actual receptive field size, *k* denote the convolution kernel size, and *r* indicate the expansion rate. The RDFBlock module can flexibly model target features at different scales by choosing different expansion rates. The pest targets are usually small and distributed in complex backgrounds for the pest detection task. The incorporation of expansion convolution effectively enlarges the receptive field and improves the model’s ability to capture background and contextual features.

The training process of the RDFBlock utilizes a multi-branch structure that includes a combination of standard convolutions and multiple sets of expansion convolutions. For input feature *x*, the module’s output can be represented as

(2) $$y=\mathrm{ReLU}\left(\mathrm{BN}\left(W_{\mathrm{std}}*x+\sum_{i=1}^{N}W_{\mathrm{dil},i}*x\right)\right)$$

$W_{\mathrm{std}}$ represents the parameters of the standard convolution, $W_{\mathrm{dil},i}$ denotes the parameters of the *i*-th dilated convolution branch, ∗ indicates the convolution operation, and BN refers to the batch normalization operation.

In order to enhance inference efficiency, RDFBlock optimizes computational efficiency by consolidating the multi-branch configuration into a single-branch form during inference, utilizing the reparameterization technique. Its weight fusion process can be expressed as

(3) $$W_{\mathrm{deploy}}=W_{\mathrm{std}}+\sum_{i=1}^{N}W_{\mathrm{dil},i}$$

**Figure 10:**
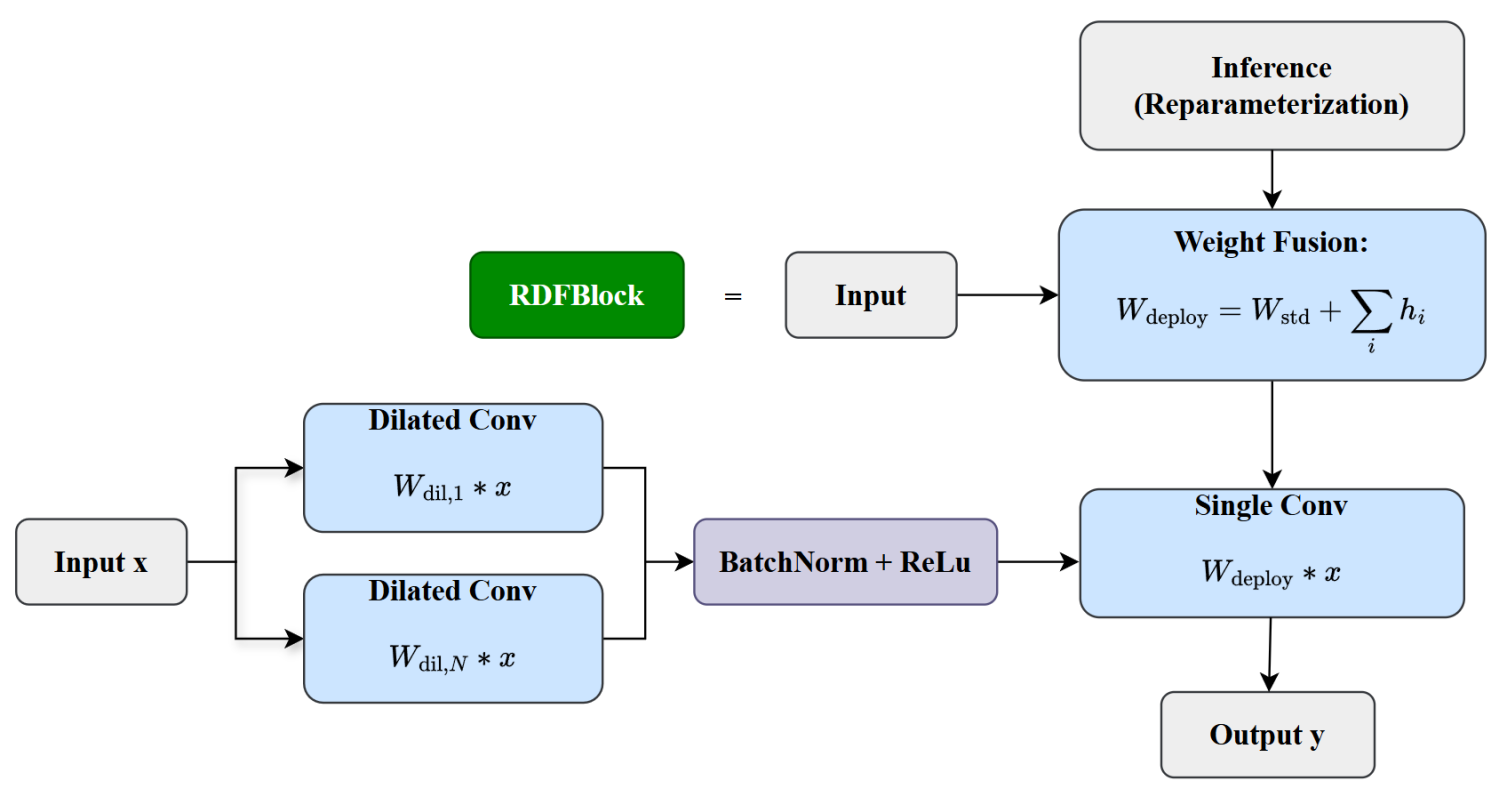
Process flow of the RDFBlock module. During training, multi-branch dilated convolutions are used to extract features, while in inference, reparameterization fuses them into a single convolution for efficiency.

The processing flow of the RDFBlock module, including its training and inference phases, is illustrated in Figure 10. This design retains the high expressiveness of the multi-branch structure during training while realizing high efficiency during inference, thus significantly reducing the computation and storage requirements in the inference phase.

The primary advantage of the proposed RDFBlock, compared to the conventional C3K2 module, lies in its enlarged receptive field and improved capability for multi-scale feature representation. These improvements render RDFBlock particularly suitable for challenging tasks such as pest detection, which require sensitivity to small-scale targets and heterogeneous backgrounds. Through the integration of dilated convolutions and structural reparameterization, RDFBlock achieves more accurate pest localization while maintaining high inference speed and computational efficiency. The RDFBlock module facilitates large-scale farmland pest surveillance and control by augmenting multi-scale feature extraction and optimizing inference efficiency in pest detection scenarios.

### 2.4. DualPathDown Module

In the original YOLO11 architecture, the downsampling process predominantly relies on either a single convolutional layer or a pooling operation to reduce the spatial resolution of feature maps. Although this strategy effectively decreases computational cost and expands the receptive field, exclusive dependence on a single operation can result in the omission of critical local and global features. Convolutional layers are proficient in extracting spatially localized information but are generally limited in modelling global context. Conversely, basic pooling operations often fail to retain fine-grained structural details, particularly along edges, which compromises feature representation fidelity. Furthermore, traditional downsampling schemes lack architectural flexibility, particularly in supporting feature map branching and multi-path processing. This deficiency hinders the model’s capacity to incorporate multi-scale contextual cues, ultimately constraining detection performance in scenarios involving small-scale objects or morphologically similar pest species.

**Figure 11:**
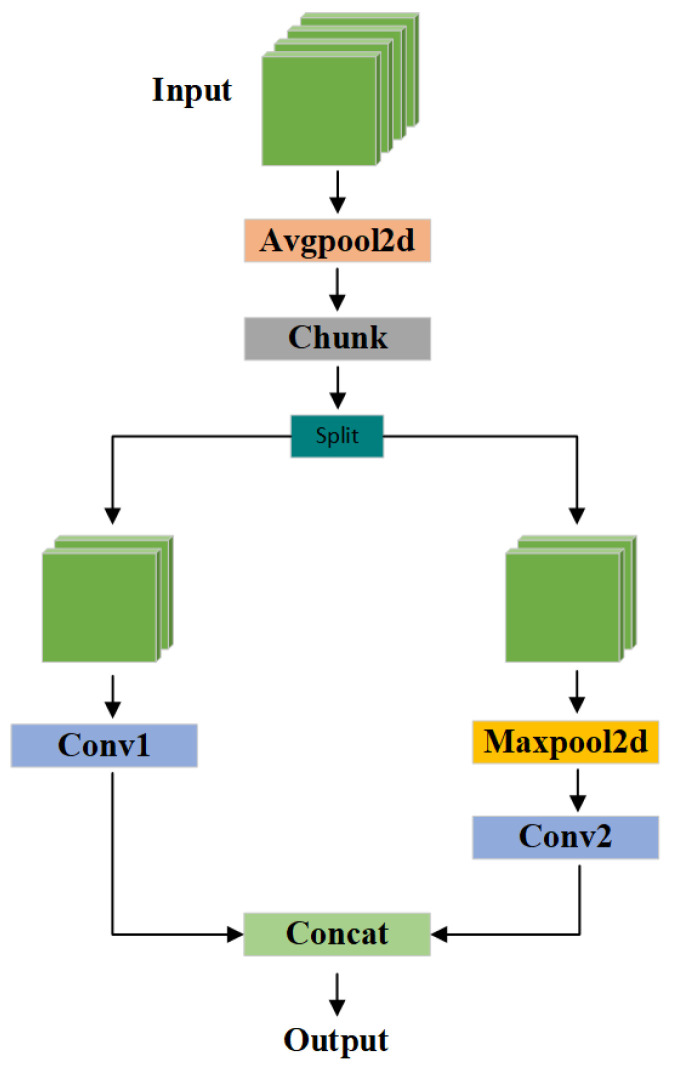
The structure of the DPDown module.

To overcome these limitations, this study proposes an improved downsampling module, referred to as DualPathDown (DPDown), designed to enhance the feature extraction capabilities of the YOLO11 framework, as depicted in Figure 11. This module is adapted from the downsampling structure of YOLOv9 [33], preserving its fundamental architecture while being reconfigured for better integration and optimized performance within the present model. In contrast to conventional single-path downsampling approaches, DPDown adopts a dual-branch architecture that enables more comprehensive feature retention. One branch utilizes a 3 × 3 convolutional layer to extract fine-grained spatial features, whereas the other combines max pooling with a 1 × 1 convolution to capture broader contextual cues. The outputs from both branches are then concatenated along the channel axis, effectively integrating localized details with high-level semantic information. This architectural enhancement increases feature diversity and representation fidelity, improving detection performance in multi-scale object recognition tasks, especially under complex environmental conditions.

The proposed DualPathDown (DPDown) module is designed to enhance the downsampling process in YOLO11 by improving feature diversity and representational capacity. It consists of two parallel branches. Initially, a global average pooling operation is applied to the input feature map *x*, and the resulting feature is partitioned along the channel dimension into two segments, $x_{1}$ and $x_{2}$. In the first branch, $x_{1}$ undergoes downsampling and local feature extraction through a 3×3 convolution layer. In parallel, the second branch processes $x_{2}$ by applying a 3 × 3 max pooling operation for downsampling, followed by a 1 × 1 convolution to reduce channel dimensionality and extract complementary features. Finally, the outputs from both branches are concatenated along the channel axis to generate the final feature representation.

The DPDown module operates as follows. The input feature *x* undergoes global average pooling:

(4) x=AvgPool(x,k=2,s=1)

Next, it is divided into two branches along the channel dimension:

(5) $$x_{1},x_{2}=\mathrm{Chunk}\left(x,2,\dim=1\right)$$

For the first branch $x_{1}$, downsampling and local feature extraction are performed via a 3×3 convolution:

(6) $$x_{1}^{\prime }=\mathrm{Conv}\left(x_{1},k=3,s=2,p=1\right)$$

For the second branch $x_{2}$, max pooling is applied for downsampling, followed by a 1×1 convolution:

(7) $$x_{2}^{\prime }=\mathrm{Conv}\left(\mathrm{MaxPool}\left(x_{2},k=3,s=2,p=1\right),k=1,s=1,p=0\right)$$

Finally, the outputs of both branches are concatenated:

(8) $$y=\mathrm{Concat}(x_{1}^{\prime },x_{2}^{\prime },\dim=1)$$

This dual-branch approach maintains downsampling efficiency while enhancing feature representation. Integrating the DPDown module enables YOLO11 to balance detection accuracy and inference speed, particularly in complex object detection scenarios.

### 2.5. Wasserstein-Weighted IoU

Optimizing the loss function is critical to improve the accuracy and stability of pest detection. Traditional methods primarily rely on Intersection over Union (IoU) to evaluate spatial consistency between predicted and ground-truth boxes [34]. However, IoU-based losses are highly sensitive to positional shifts, where minor deviations significantly impact gradient stability, particularly in pest detection scenarios with irregular shapes, small objects, and complex backgrounds, leading to reduced localization accuracy and model reliability.

To mitigate these issues, this study proposes Wasserstein-Weighted IoU (WWIoU), a novel loss function improving robustness in pest detection. WWIoU integrates Complete IoU (CIoU) [35], which enables accurate bounding-box regression, with Normalized Wasserstein Distance (NWD) [36], which captures geometric relationships between bounding-boxes. This integration enhances the precision of similarity measurement, reduces sensitivity to minor positional deviations, and improves spatial alignment in object detection tasks.

The core contribution of WWIoU lies in the introduction of NWD to redefine the similarity measurement between bounding-boxes. In this formulation, both the predicted and ground-truth boxes are modeled as two-dimensional Gaussian distributions, denoted as $N_{g}\left(\mu_{g},\Sigma_{g}\right)$ and $N_{t}\left(\mu_{t},\Sigma_{t}\right)$, respectively. Here, $\mu_{g}$ and $\mu_{t}$ represent the centroid coordinates, while $\Sigma_{g}$ and $\Sigma_{t}$ denote the covariance matrices, characterizing the scale and shape of each bounding-box. By incorporating this probabilistic representation, WWIoU captures spatial and scale discrepancies, resulting in a more stable and precise regression process.

Based on this, the geometric difference between two Gaussian distributions is measured by computing the second-order Wasserstein distance, defined as follows:

(9) $$W_{2}^{2}\left(N_{g},N_{t}\right)={∥\mu_{g}-\mu_{t}∥}^{2}+\mathrm{Tr}\left(\Sigma_{g}+\Sigma_{t}-2{\left(\Sigma_{t}^{1/2}\Sigma_{g}\Sigma_{t}^{1/2}\right)}^{1/2}\right)$$

The first term, $|\mu_{g}-\mu_{t}{|}_{2}^{2}$, quantifies the squared Euclidean distance between the centroids of the predicted and ground-truth frames. The second term, $\mathrm{Tr}(\Sigma_{g}+\Sigma_{t}-2{\left(\Sigma_{t}^{1/2}\Sigma_{g}\Sigma_{t}^{1/2}\right)}^{1/2})$, characterizes the covariance discrepancy, that is, the difference in the distributions of shape and scale between the two frames. By integrating these two components, the second-order Wasserstein distance provides a comprehensive characterization of the geometric relationship between the predicted and ground-truth frames, thereby offering richer global information for bounding-box optimization.

To enhance the applicability of the Wasserstein distance, we normalize it to obtain the Normalized Wasserstein Distance. The normalized formula is

(10) $$\mathrm{NWD}\left(N_{g},N_{t}\right)=e^{-\frac{W_{2}^{2}\left(N_{g},N_{t}\right)}{c}}$$

where *c* is a normalization constant used to regulate the distance range at different scales. Through the normalization process, NWD maintains a stable similarity metric capability in target detection scenarios with different scales and complex distributions. Unlike traditional IoU, NWD accurately reflects the geometric distribution relationship between bounding-boxes and has stronger robustness to positional offsets and scale changes, thus effectively mitigating the instability caused by IoU sensitivity throughout the training process.

In order to further refine the model performance, WWIoU organically integrates NWD with CIoU, which can accurately optimize the target frame’s local characteristics by combining the IoUs, centroid distances, and aspect ratio differences. Its expression is

(11) $$L_{\mathrm{CIoU}}=1-\mathrm{IoU}+\frac{∥{\mathrm{center}}_{\mathrm{pred}}-{\mathrm{center}}_{\mathrm{true}}{∥}_{2}^{2}}{d^{2}}+\alpha v$$

where IoU represents the ratio of intersection over union between the predicted and ground-truth bounding-boxes, $∥{\mathrm{center}}_{\mathrm{pred}}-{\mathrm{center}}_{\mathrm{true}}{∥}_{2}^{2}$ quantifies the squared Euclidean distance between the centers of the predicted and actual bounding-boxes, *d* indicates the diagonal length of the smallest enclosing box that contains both predicted and ground-truth frames, and αv is used to ensure consistency in the aspect ratio between these frames.

The WWIoU loss function serves as an integrated metric that efficiently combines the strengths of NWD and CIoU. It is mathematically formulated as follows:

(12) $$L_{\mathrm{WWIoU}}=r\cdot L_{\mathrm{CIoU}}+\left(1-r\right)\cdot L_{\mathrm{NWD}}$$

In Equation (12), *r* is a weighting coefficient used to balance the contributions of CIoU and NWD in the loss function. By leveraging this weighted fusion, WWIoU achieves complementary advantages between global geometric modeling and local bounding-box optimization, leading to notable enhancements in the model’s robustness and accuracy in complex scenarios.

In pest detection tasks, WWIoU demonstrates superior performance by integrating Normalized Wasserstein Distance (NWD) to capture the geometric distribution of target boxes more effectively, particularly for irregular shapes or severe overlaps. Meanwhile, Complete IoU (CIoU) enhances bounding-box regression by improving overlap and positional accuracy. Beyond detection precision, WWIoU also improves training stability. Leveraging NWD’s robustness to positional offsets and scale variations mitigates convergence issues caused by sample imbalance or poor anchor matching. Additionally, its normalization ensures scale-invariant consistency, enhancing adaptability to varying object sizes and complex detection scenarios. Overall, WWIoU provides an efficient, precise, and robust solution for pest detection.

## 3. Experiments

### 3.1. Experimental Setting and Assessment Indicators

In this study, the initial learning rate is set to 0.01, the batch size is 32, and the number of epochs is 200. The experiments are conducted in a deep learning environment based on the Ubuntu 22.04.4 LTS operating system. The specific hardware configuration includes an NVIDIA RTX 3090 GPU. The experimental environment configurations are detailed in Table 1, and the hyperparameter settings are listed in Table 2.

To comprehensively evaluate model performance, this study employs several metrics: precision (P), recall (R), mean average precision (mAP), number of parameters (Params), and computational complexity (FLOPs). Precision (P) is described as the proportion of true positive predictions to the total number of positive predictions, as expressed in the formula below:

(13) $$P=\frac{TP}{TP+FP}\times 100\%$$

Recall (R) measures the model’s ability to capture the target samples, defined as the ratio of correctly predicted positive samples to all true positive samples, as given by the following formula:

(14) $$R=\frac{TP}{TP+FN}\times 100\%$$

Here, TP, FP, and FN represent the number of true positives, false positives, and false negatives, respectively.

The mean average precision (mAP) is a key metric for evaluating the overall detection performance of the model, which is calculated based on the average precision (AP) of each category, and the AP is calculated by the area under the precision–recall (P-R) curve with the following formula:

(15) $$AP=\int_{0}^{1}P\left(R\right)dR,\;mAP=\frac{1}{N}\sum_{i=1}^{N}AP_{i}$$

The structural complexity of a model is typically evaluated using two metrics: the number of parameters (Params) and floating-point operations (FLOPs). Params indicate the total trainable parameters, where fewer parameters reduce storage and deployment costs, facilitating use in embedded systems. FLOPs measure computational efficiency per image, with lower values minimizing computational overhead, enhancing inference efficiency on resource-limited hardware.

### 3.2. Experimental Results of RDW-YOLO Model

**Figure 12:**
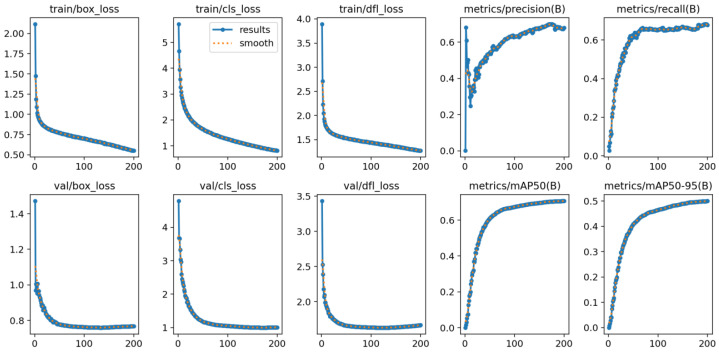
Performance of RDW-YOLO model on the enhanced IP02 dataset.

Figure 12 demonstrates the performance of the RDW-YOLO model on the enhanced IP02 dataset. The trend in the figure clearly shows that, as the training process advances, various loss metrics and generation counts gradually change. Similarly, the bounding-box precision, recall, and mAP metrics are adjusted accordingly. These modifications highlight the model’s effectiveness in detection tasks.

**Figure 13:**
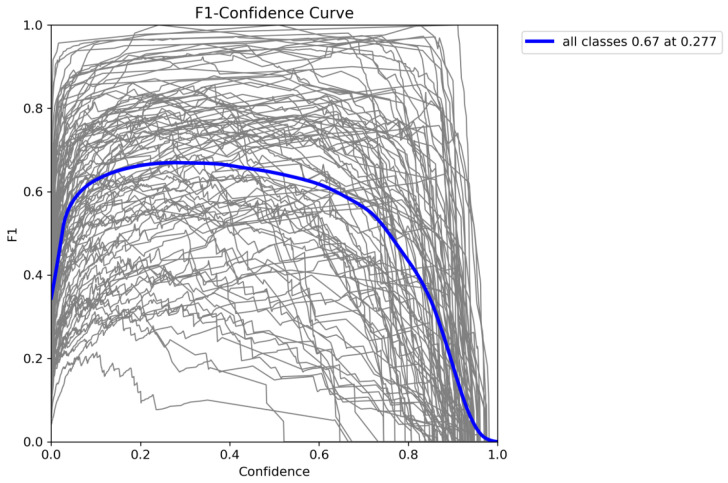
F1 score curves for each pest category under different confidence thresholds.

To better evaluate the model’s ability to identify pests at different confidence levels, Figure 13 presents the F1 score curves for various pest categories. The F1 score, which balances precision and recall, effectively reflects the model’s performance in pest detection.

**Figure 14:**
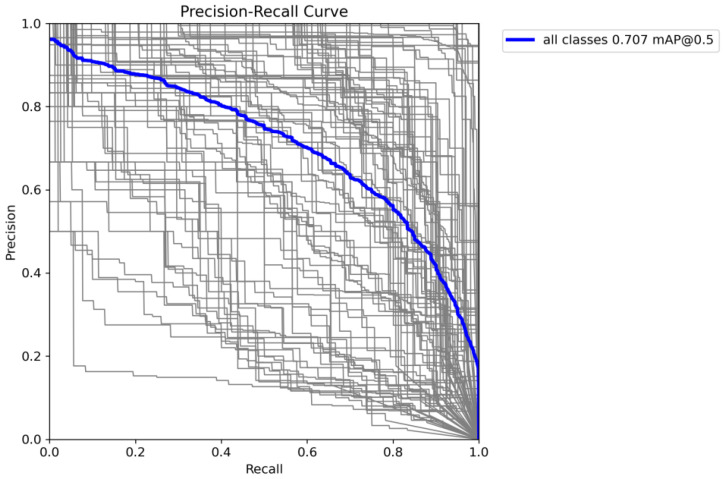
The precision–recall curve for the RDW-YOLO model.

Figure 14 illustrates the precision–recall curve for the RDW-YOLO model in pest detection, from which the change of the model’s precision rate under different recall rates can be observed. The curve exhibits a smooth decreasing trend, indicating that the model effectively maintains precision stability while achieving high recall rates. The model demonstrates excellent performance in pest target detection, with an mAP@0.5 of 0.707.

This performance highlights the RDW-YOLO model’s advantage in balancing precision and recall, enabling it to capture pest targets effectively while reducing false positives and missed detections.

### 3.3. Ablation Experiments

**Figure 15:**
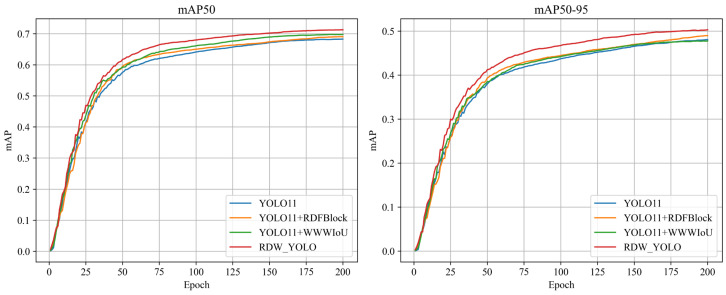
Contribution of each component to overall network performance: ablation experiment results.

In order to evaluate the impact of each component in the proposed optimization algorithm on the overall network performance, we conducted targeted independent tests to assess the effectiveness of each component. Figure 15 presents a detailed overview of the experimental results.

As demonstrated in Table 3, the proposed improvement modules in this study consistently outperform the original YOLO11 algorithm across all evaluation metrics, including mAP@0.5 and mAP@0.5:0.95. A series of ablation experiments were conducted to assess each enhancement’s effectiveness systematically.

The RDFBlock module was integrated into the backbone network to enhance feature extraction through multi-branch dilated convolutions. This modification led to a 0.9% increase in mAP@0.5, underscoring its effectiveness in capturing complex feature representations and improving the model’s overall expressiveness. The DPDown module was introduced into the downsampling process to optimize feature extraction efficiency by leveraging a hybrid pooling and convolution mechanism. This enhancement facilitated a more robust adaptation to multi-scale targets, yielding a 0.8% improvement in mAP@0.5. The original regression mechanism was replaced with the WWIoU loss function to improve localization accuracy and bounding-box regression performance. This adjustment significantly refined the model’s capability to align predicted bounding-boxes with ground-truth objects, resulting in a 1.5% increase in mAP@0.5.

When RDFBlock and DPDown are integrated, the model achieves an mAP@0.5 of 69.6%, exceeding the performance of each module used independently. This outcome underscores the complementary advantages of enhanced feature extraction and improved multi-scale representation. The combination of RDFBlock and WWIoU yields a slightly higher mAP@0.5 of 69.8%, accompanied by increased recall, indicating that the joint optimization of feature representation and localization accuracy is particularly effective for detecting small and complex pest targets. When DPDown is combined with WWIoU, the model maintains stable performance, reflecting the synergy between efficient downsampling and precise bounding-box regression. Notably, the integration of all three modules results in the highest overall performance, with an mAP@0.5 of 71.3% and mAP@0.5:0.95 of 50.0%. This demonstrates the additive and reinforcing effects of the proposed architectural enhancements. These results collectively validate that the proposed modules offer individual benefits and interact synergistically to significantly improve detection accuracy, generalization, and robustness in complex agricultural scenarios.

The integration of the RDFBlock, DPDown, and WWIoU modules into the YOLO11 framework yields a cumulative improvement of 3.1% in mAP@0.5, increasing from 68.2% to 71.3%, as shown in Table 3. This gain is meaningful in agricultural scenarios, as it helps reduce false positives and missed detections, particularly among visually similar pest species. Moreover, the enhanced detection accuracy enables early identification of pests at their larval stages, facilitating timely intervention and preventing further crop damage.

Computationally, the proposed architecture reduces FLOPs by approximately 1 G, from 6.6 G to 5.6 G. This combination of improved accuracy and reduced computational complexity enhances the model’s suitability for deployment on embedded systems with limited hardware resources.

Overall, this improvement reflects a favorable cost–benefit trade-off. Better detection accuracy supports more precise pest control interventions, while lower complexity enables real-time processing and wider applicability in intelligent agricultural systems.

### 3.4. Target Comparison Experiments

Comparison experiments with mainstream target detection algorithms were performed to assess the effectiveness of the proposed improved algorithm. Table 4 summarizes the performance of each model in terms of precision (P), recall (R), mAP@0.5, mAP@0.5:0.95, parameter counts (Params), and computational complexity (FLOPs).

From Table 4, the proposed model demonstrates superior performance in both detection accuracy and computational efficiency compared to existing models. Specifically, it achieves a 3.1% increase in mAP@0.5 and a 2% increase in mAP@0.5:0.95 over YOLO11, while simultaneously reducing the number of parameters from 5.6 M to 4.7 M and FLOPs from 6.6 G to 5.6 G. This improvement highlights the model’s ability to maintain high accuracy with significantly lower computational costs.

Compared to YOLOv10, the proposed model improves mAP@0.5 by 4% and mAP@0.5:0.95 by 3%, with parameters reduced by 1.3 M and FLOPs by 1.3 G, demonstrating a favorable balance between accuracy and efficiency. Against YOLOv8, it achieves a 3.3% improvement in mAP@0.5 and a 2.2% increase in mAP@0.5:0.95 while reducing the parameter count by 2.2 M and FLOPs by 4 G.

These results suggest that the proposed model significantly enhances detection accuracy while substantially lowering computational costs, making it a practical and effective solution for resource-constrained applications.

### 3.5. Dataset Detection Results

**Figure 16:**
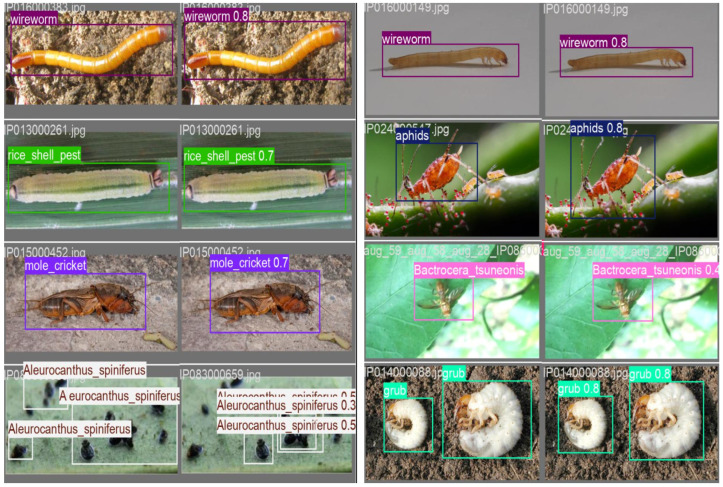
Detection results of the YOLO model for various pest species.

Based on the detection results shown in Figure 16, the model demonstrates strong performance in identifying various types of pests. Most targets are accurately detected and correctly localized, with high confidence scores. For instance, pests such as *wireworm*, *rice shell pest*, *mole cricket*, and *aphids* are properly labeled, with confidence scores generally exceeding 0.7. This indicates that the model achieves reliable recognition for these categories.

In terms of robustness, the model performs well across different environmental conditions. For example, *wireworm* is accurately detected in both soil environments and experimental settings (white background), without noticeable false positives or missed detections. This stability suggests that the model extracts consistent features for this pest type and is not significantly affected by background variations. Similarly, the fruit fly pest *Bactrocera tsuneonis* is correctly identified under varying lighting conditions, further demonstrating the model’s strong generalization ability.

In multi-object detection tasks, the model effectively identifies multiple instances within a single image. For example, when processing images containing *Aleurocanthus spiniferus*, it successfully localizes several individuals and assigns confidence scores. However, these scores exhibit notable variation, typically ranging from 0.3 to 0.5, indicating instability in classifying this pest species. This inconsistency may result from a limited number of annotated training samples or the small size and low visual saliency of the targets.

The detection results indicate that the model performs effectively in pest detection tasks, reliably identifying multiple targets across diverse backgrounds and lighting conditions. However, for smaller pest species such as *Aleurocanthus spiniferus*, the relatively low confidence scores highlight the need for additional dataset refinement or hyperparameter tuning to improve recognition reliability.

**Figure 17:**
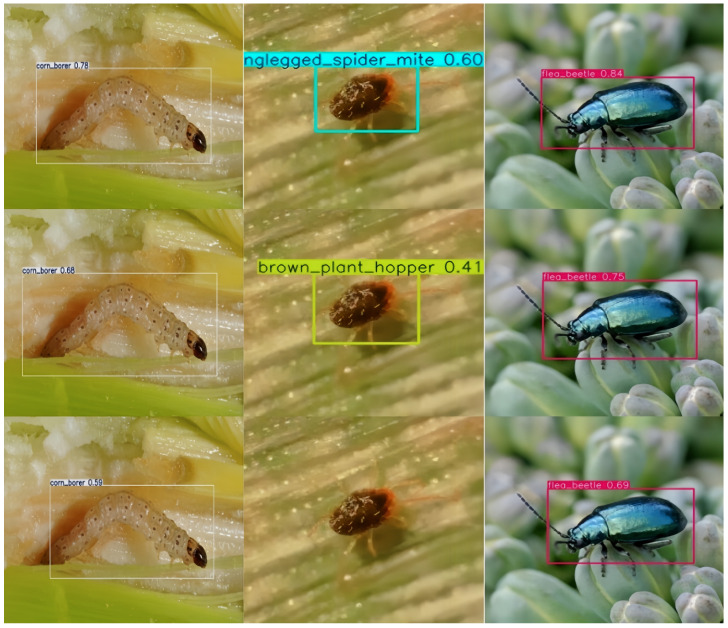
Detection comparison: The first column shows RDW-YOLO results, the middle column shows YOLO11 results, and the third column shows YOLOv8 results.

To assess the detection performance of the proposed algorithm, experiments were conducted on randomly sampled images from the dataset. The final detection results were compared between the original YOLO11 algorithm and the improved method. Figure 17 presents a visual comparison of the outcomes. As shown in the figure, the proposed RDW-YOLO algorithm significantly improves detection accuracy compared to the original models.

For *corn borer* detection, RDW-YOLO achieves a confidence score of 0.78, outperforming YOLO11 (0.68) and YOLOv8 (0.59). This highlights RDW-YOLO’s superior accuracy and stability in detecting large targets, making it well suited for applications requiring high confidence levels. In contrast, YOLO11 and YOLOv8 exhibit weaker performance, particularly YOLOv8, which may require further optimization for this type of detection.

In *spider mite* detection, RDW-YOLO achieves a confidence score of 0.60, accurately identifying *monopodal spider mites*. In comparison, YOLO11 misclassifies the *brown planthopper* with a confidence score of 0.41, showing its limitations in small target detection. YOLOv8 fails to recognize the category, further demonstrating its weaker performance in this scenario. These results underline RDW-YOLO’s superior classification and detection accuracy for small targets.

For *jumping armor* detection, RDW-YOLO achieves the highest confidence score of 0.84, surpassing YOLO11 (0.75) and YOLOv8 (0.69). This highlights its suitability for high-confidence small target detection. While YOLOv8 demonstrates some competitiveness in this scenario, its overall lower confidence score reflects its relatively weaker detection capability.

Overall, RDW-YOLO excels in detection stability and classification accuracy, especially in high-precision scenarios. While YOLO11 and YOLOv8 show flexibility in multi-target detection, they require further improvement in confidence and accuracy.

## 4. Discussion

Despite RDW-YOLO’s promising performance on curated datasets, RDW-YOLO exhibited limitations in complex and unstructured agricultural environments, such as densely vegetated fields, occluded or overlapping pest instances, and highly variable illumination. These challenges highlight the need for further investigation into the model’s robustness and generalizability. Future work should explore the adaptability of RDW-YOLO across diverse crop types, lighting conditions, and ecological regions, potentially leveraging transfer learning and domain adaptation techniques. To facilitate this, future research efforts will aim to expand the pest image dataset to include a broader range of environmental contexts, seasonal variations, and geographic distributions. Additionally, incorporating multimodal inputs such as infrared or hyperspectral data may further enhance detection performance under challenging conditions.

Moreover, while RDW-YOLO demonstrates significant improvements in detection accuracy and computational efficiency, realizing its full potential in practical agricultural systems necessitates further exploration. A key step involves evaluating the model’s deployment on edge computing platforms such as NVIDIA Jetson or Raspberry Pi, which support decentralized, low-latency inference suitable for in-field decision-making. Building upon this computational infrastructure, integrating RDW-YOLO with unmanned aerial vehicles (UAVs) presents a promising direction for achieving scalable, mobile pest surveillance. Such integration could enable continuous, wide-area monitoring with reduced labor input across heterogeneous agricultural landscapes.

RDW-YOLO offers an effective and efficient solution for intelligent pest detection. However, translating these algorithmic advances into real-world impact will require sustained efforts in scalable deployment, cross-domain generalization, and ecosystem-specific customization. These future developments will be instrumental in driving the adoption of sustainable, technology-enabled solutions for precision agriculture and pest management.

## 5. Conclusions

In this study, we propose an improved pest and disease detection algorithm, RDW-YOLO, based on the YOLO11 framework. By integrating the RDFBlock, DPDown module, and WWIoU loss function, the proposed method enhances model performance in terms of three key aspects: feature extraction capability, downsampling efficiency, and bounding-box regression accuracy. Experimental results on the enhanced IP102 dataset demonstrate that RDW-YOLO achieves an mAP@0.5 of 71.3% and an mAP@0.5:0.95 of 50.0%, outperforming mainstream detection algorithms. Furthermore, RDW-YOLO significantly reduces model parameters and computational complexity, highlighting its potential for deployment in resource-constrained environments.

Despite these promising results, certain challenges remain, particularly in adapting the model to complex real-world scenarios. These include varying illumination conditions, densely distributed targets, and accurate detection of small objects. Future work will focus on enhancing the model’s robustness under such conditions and adaptability to varying object scales and occlusions. Additionally, with the advancement of agricultural intelligence, efforts will be directed toward further model lightweighting and optimizing real-time performance to ensure efficient operation on edge devices.

Moreover, future research may consider expanding the dataset diversity and incorporating multimodal data such as infrared and spectral imagery to improve detection performance in low-contrast or complex environments. With integration into broader agricultural IoT systems, RDW-YOLO holds significant promise as a core component of intelligent pest and disease monitoring and management, contributing to precision agriculture, improved crop yields, and enhanced food security.

## Figures and Tables

**Table 1:** Experimental environment parameters.

Component	Name/Value
Operating System	Ubuntu 22.04
GPU	NVIDIA RTX 3090
Video Memory	24G
Training Acceleration	CUDA 12.1
Programming Language	Python 3.11
Deep Learning Framework	PyTorch 2.1.0

**Table 2:** Hyperparameter settings.

Component	Name/Value
Epochs	200
Batch	32
AdamW Learning Rate	0.01
Workers	4
Momentum	0.937
Patience	100

**Table 3:** Baseline-based ablation experiments. The ✓ indicate the presence of the corresponding component in the model configuration.

RDFBlock	DPDown	WWIoU	mAP (0.5)	mAP (0.5:0.95)	P	R
			68.2%	48%	67.4%	65%
✓			69.1%	48.8%	66.2%	66.8%
	✓		68.8%	48.6%	68.4%	63.7%
		✓	69.8%	47.7%	66.2%	64.3%
✓		✓	69.6%	48.9%	66.2%	66.5%
	✓	✓	69.8%	48.9%	67.7%	66.2%
✓	✓		69.7%	49.3%	65.9%	67%
✓	✓	✓	71.3%	50%	67.7%	68%

**Table 4:** Comparison of different models in the experiment.

Methods	mAP (0.5)	mAP (0.5:0.95)	P	R	ModelSize	FLOPs
Faster-RCNN [37]	50.3%	38.2%	42.4%	54.8%	41.8 M	135 G
Cascade R-CNN [38]	51.1%	41.8%	48.3%	55.2%	77.3 M	268 G
DETR [39]	49.4%	30.3%	64.2%	48.6%	53.2 M	184 G
YOLOv5	66.5%	45.4%	63.8%	63.6%	5.9 M	8.6 G
YOLOv6	64.4%	44.7%	61.2%	61.9%	9.5 M	13.7 G
YOLOv8	68%	47.8%	66.1%	64.2%	6.9 M	9.6 G
YOLOv10	67.3%	47%	63.1%	64%	6 M	6.9 G
YOLO11	68.2%	48%	67.4%	65%	5.6 M	6.6 G
**ours**	**71.3%**	**50%**	**67.7%**	**68%**	**4.7 M**	**5.6 G**

## Data Availability

The original contributions presented in this study are included in the article. Further inquiries can be directed to the corresponding author.

## Abbreviations

The following abbreviations are used in this manuscript:

YOLO11	You Only Look Once version 11
C2PSA	Cross Stage Partial with Pyramid Squeeze Attention
DWConv	Depthwise Convolution
C3	CSP Bottleneck with three convolutions
NWD	Normalized Wasserstein Distance
IoU	Intersection over Union
WWIoU	Wise-Wasserstein IoU
mAP	Mean Average Precision
mAP50	MAP values at the 50% IoU threshold
mAP50:95	MAP values in the 50–95% IoU threshold range
CNN	Convolutional neural network
Faster R-CNNs	Faster region-based convolutional neural networks
DF Loss	Distribution focal loss
CIOU	Complete Intersection over Union
GFLOPs	Giga Floating-Point Operations Per second
